# Molecular detection and clinical characteristics of *Bartonella bacilliformis*, *Leptospira* spp., and *Rickettsia* spp. in the Southeastern Peruvian Amazon basin

**DOI:** 10.1186/s12879-018-3541-7

**Published:** 2018-12-04

**Authors:** Fiorella Ricapa-Antay, Katia Diaz-Melon, Wilmer Silva-Caso, Luis J. del Valle, Miguel Angel Aguilar-Luis, Fernando Vasquez-Achaya, Carlos Palomares-Reyes, Pablo Weilg, Dongmei Li, Carlos Manrique, Juana del Valle-Mendoza

**Affiliations:** 1 School of Medicine. Faculty of Health Sciences. Research and Innovation Centre of the Faculty of Health Sciences, Universidad Peruana de Ciencias Aplicadas, Lima, Peru; 2 Laboratorio de Biología Molecular. Instituto de Investigación Nutricional, Lima, Peru; 3 Barcelona Research Center for Multiscale Science and Engineering, Departament d’Enginyeria Química, EEBE, Universidad Politécnica de Catalunya (UPC), Barcelona Tech, Barcelona, Spain; 4 Instituto de Investigación de Enfermedades Infecciosas, Lima, Peru; 5 State Key Laboratory for Infectious Disease Prevention and Control, Collaborative Innovation Center for Diagnosis and Treatment of Infectious Diseases, National Institute for Communicable Disease Control and Prevention, Chinese Center for Disease Control and Prevention, Changping, Beijing China; 6 Dirección Regional de Salud Madre de Dios (DIRESA-Madre de Dios), Puerto Maldonado, Madre de Dios Peru

**Keywords:** *Bartonella bacilliformis*, Rickettsia, Leptospira, Acute febrile illness, Peru

## Abstract

**Background:**

Acute febrile illness (AFI) represent a significant health challenge in the Peruvian Amazon basin population due to their diverse etiologies and the unavailability of specific on-site diagnostic methods, resulting in underreporting of cases. In Peru, one of the most endemic regions to dengue and leptospirosis is Madre de Dios, a region also endemic to emergent bacterial etiologic agents of AFI, such as bartonellosis and rickettsiosis, whose prevalence is usually underreported.

We aimed to molecularly identify the presence of *Leptospira* spp*.*, *Bartonella bacilliformis*, and *Rickettsia* spp*.* by Polymerase Chain Reaction in serum samples from patients with AFI from Puerto Maldonado-Madre de Dios in Peru.

**Methods:**

Serum samples from patients with acute febrile illness were analyzed by real-time PCR for detecting the presence of *Bartonella bacilliformis*, *Leptospira* spp. and *Rickettsia* spp.

**Results:**

*Bartonella bacilliformis* was the most prevalent bacteria identified in 21.6% (30/139) of the samples, followed by *Leptospira* spp*.* in 11.5% (16/139) and *Rickettsia* spp*.* in 6.5% (9/139) of the samples. No co-infections were observed between these bacteria. The most frequent symptoms associated with fever among all groups, were headaches, myalgias, and arthralgias. We found no statistically significant differences in the clinical presentation between patients infected with each bacterium.

**Conclusions:**

In a previous study, we shown the presence of dengue, chikungunya, Zika and oropouche virus. We were able to identify these pathogens in 29.5% of all the samples, with chikungunya and OROV as the most frequently found in 9.4 and 8.6% of all the samples, respectively. In this study we show that *B. bacilliformis* (21.6%), *Leptospira* spp. (11.5%) and *Rickettsia* spp. (6.5%) accounted for the main etiologies of AFI in samples from Puerto Maldonado-Madre de Dios, Perú. Our analysis of their clinical presentation, further shows the importance of implementing more sensitive and specific on-site diagnostic tools in the national surveillance programs.This study confirms that the un-specificity of signs and symptoms is not only associated with arboviral infections, but also with the clinical presentation of endemic bacterial infections.

## Introduction

Acute febrile illness (AFI) is one of the most common syndromes in the tropics and subtropics associated with different viral, parasites and bacterial etiologies [1, 2]. The clinical presentation of these infections shares many symptoms and are, therefore, non-specific to the etiologic agent [3]. This poses a diagnostic challenge among on-site rural clinicians that lack specific diagnostic tools which in turn has led to under-reporting of these etiological agents in low to middle-income countries in South America [1, 4, 5].

In recent years, Peru has experienced a resurgence of arthropod-borne arboviral diseases such as dengue, chikungunya, Zika, and oropouche, as well as leptospirosis and rickettsiosis [6–10]. Previous reports have shown that dengue and leptospirosis are the most common causes of AFI in Peru [5]. However, in the Peruvian Amazon basin, dengue virus has been reported to account for the etiology of 6-26% of patients with AFI and other co-circulating pathogens are hypothesized to have higher prevalence but have low laboratory confirmation rates [4, 5]. There is evidence of the presence of Orientia spp., the etiological agent of scrub typhus, as a probable cause of AFI in the Peruvian Amazon. Although this has not been confirmed by molecular methods, the serological evidence and the multiple possible vectors suggest the presence of this pathogen in tropical areas of Peru [11].

Madre de Dios is located in the southern region of Peru, within the Amazon Basin. It is the third most endemic region to dengue fever; as well as for other circulating arboviruses including chikungunya and oropouche virus [6, 7]. Furthermore, the introduction of novel zika virus infections in this region was described in 2017, according to national epidemiologic reports [12–16]. Madre de Dios is also the most endemic region in Peru for leptospirosis, with a total of 1001 cases reported during 2016 [17]. Rickettsial diseases are also neglected infections and a potential cause of AFI that remains under-reported due to the empirical protocol treatment and limited access to diagnostic tools in isolated localities within the Peruvian Amazon Basin [10, 18].

*Bartonella bacilliformis* is the etiological agent of Carrion's disease, another common, widespread cause of acute febrile illness endemic to at least 14 territories of Peru, including Madre de Dios [19]. The endemicity of this infection is mostly restricted to areas of the Andean valleys in Peru, however, following the initial identification of Carrion’s disease in Madre de Dios during 2014, a pattern of increasing yearly prevalence has been observed and this etiology has become an important cause of AFI [20–23]. Moreover, co-infections between *B. bacilliformis* and *Leptospira* spp. have demonstrated an increase in the clinical severity of the disease [24].

In a previous study [25], we aimed to molecularly detect the presence of dengue, chikungunya, Zika and oropouche virus among patients with AFI in Madre de Dios. We were able to identify these pathogens in 29.5% of all the samples, with chikungunya and OROV as the most frequently found in 9.4% and 8.6% of all the samples, respectively. We hypothesized that this low detection rate was due to the implication of other etiological agents responsible for AFI among these patients.

As a branch of our previous research, this study aims to molecularly identify endemic bacterial etiologies of AFI including *Leptospira* spp, *Rickettsia* spp, and *Bartonella bacilliformis* in the serum samples of patients from our previous study in Madre de Dios and to describe their clinical and epidemiological characteristics.

## Materials and methods

### Place of study

This is a consecutive cross-sectional study that was conducted in Puerto Maldonado between January and March of 2016 within nine primary health care centers in coordination with the *“Regional Directive of Health in Madre de Dios.*” Puerto Maldonado is the capital of Madre de Dios, located in the Amazon rainforest at 308 meters above sea level. Patients that fulfilled the inclusion criteria were recruited for the molecular detection of *Bartonella bacilliformis*, *Leptospira* spp. and *Rickettsia* spp*.*

### Study subjects

The inclusion criteria were patients who presented to Internal Medicine-Pediatrics outpatient health centers with acute febrile illness, defined as an axillary temperature greater than or equal to 38°C within at least 7 days prior to consultation without an identifiable source of infection. The following signs and symptoms were assessed and recorded by the attending physician in a standardized questionnaire: headache, muscle pain, joint pain, loss of appetite, retro-ocular pain, nausea, vomiting, chills, dizziness, rash, sore throat, photophobia, abdominal pain, cough, pallor, diarrhea, conjunctival injection, rhinorrhea, shortness of breath, dysuria, fatigue, jaundice and seizures.

Exclusion criteria were patients who had received treatment before the consult, patients with an incomplete record of their medical information and patients with an identifiable source of infection, such upper or lower respiratory tract infections, urinary tract infections, among others.

### Ethics statement

This study has been approved by two independent Ethics Committees from *Universidad Peruana de Ciencias Aplicadas* and *Hospital Regional de Cajamarca.* A written informed consent was signed before enrollment; for participants under 18 years old the informed consent was signed by their respective guardians before enrollment.

### Samples

A total of 139 patients were sampled and blood was collected by using Vacuette® TUBE Serum Separator Clot Activator (Vacuette, Greiner Bio-One, Kremsmünster, Austria). After collection, all the samples were stored at -80°C and transported to Lima (Peru) under standardized frozen conditions to perform molecular assays. In a previous study [25], in these samples we detect the presence of dengue, chikungunya, Zika and oropouche.

### DNA extraction

DNA extraction was performed following the instructions of a commercial extraction kit (High Pure Kit Preparation template, Roche Applied Science, Mannheim, Germany) using 200 μl of the collected samples. Bacterial DNA obtained after extraction was eluted in 100 μl of nuclease-free water and then processed or stored at -20°C until use.

### PCR amplification

#### Real-time PCR assay detection of *Bartonella bacilliformis*, *Leptospira* spp. and *Rickettsia* spp.

The PCR was performed using specific primers and probe for species-specific gene of *Bartonella bacilliformis* [26], gene PanR8 of *Rickettsia* spp. [27] and gene LipL32 of *Leptospira* spp. [28] as previously described. Each reaction contained 5 μl of template DNA and 15 μl of PCR master mix (FastStar PCR Master, Roche Diagnostic, Germany) including 1 μl (10 μM) each of forward and reverse primers and 1.2 μl (10 μM) Taqman probe. The qPCR conditions were 95°C for 2 minutes, 55 cycles of 3 seconds at 95°C, 30 seconds at 55°C and 10 seconds at 72°C.

For *B. bacilliformis*, the collection strain (CIP 57.19, NCTC 12135) was used as the positive control, and the positive control for *Leptospira* spp. and *Rickettsia* spp*.* were used strains of *Leptospira noguchii* and *Rickettsia typhi* provided by the microbiology laboratory of Institute of Nutritional Research in Lima, Peru. A PCR reaction without template DNA was used as the negative control in all the cases. As an internal control, a PCR targeting the gene encoding human beta-globin was included in each PCR series to rule out the possibility of PCR inhibition caused by inhibitory molecules still present in the sample after extraction and purification of the DNA.

### Data analysis

Qualitative variables were reported as frequencies and percentages. All analyses were processed with the IBM Statistical Package for the Social Sciences (SPSS) software version 21.0 (SPSS, Chicago, IL, USA). The confidence interval to the 95% was estimated for each frequency or odds, and two frequencies or odds were compared with the odds ratio. Chi-square test (|^2^) was used to estimate differences statistical (*p* δ 0.05). Pearson correlation was used to determined statistical association.

## Results

### Demographic characteristics

**Fig. 1:**
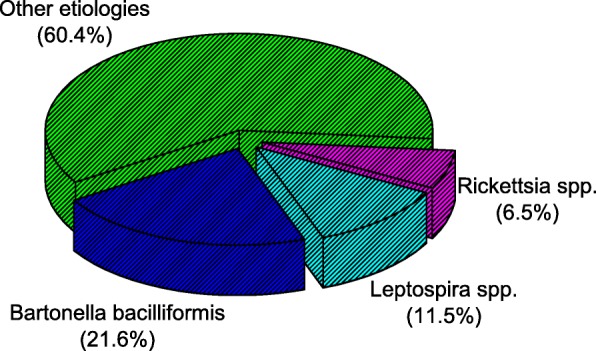
Frequency of *Bartonella bacilliformis, Leptospira* spp. and *Rickettsia* spp*.* as etiological agents of AFI in Puerto Maldonado, Madre de Dios, Peru

A total of 139 samples from patients with acute febrile illness (AFI) from Puerto Maldonado-Madre de Dios were included and molecularly analyzed in this study. Figure 1 shows the etiological detection distribution and demonstrates the significantly higher frequency of *Bartonella bacilliformis* (21.6%, *p*<0.05) and *Leptospira* spp*.* (11.5%, *p*<0.05) as etiologic agents of AFI with odds of 0.275 (CI95%: 0.184-0.411) and 0.130 (CI95%: 0.077-0.218), respectively. Additionally, *Rickettsia* spp*.* was the less frequent bacterial etiologic agent identified in 6.5% (*p*>0.05) of all cases with odds of 0.069 (CI95%: 0.032-0.134). Thus, these three bacterial etiological agents together were responsible for 40% of the cases of AFI in the population studied.

**Fig. 2:**
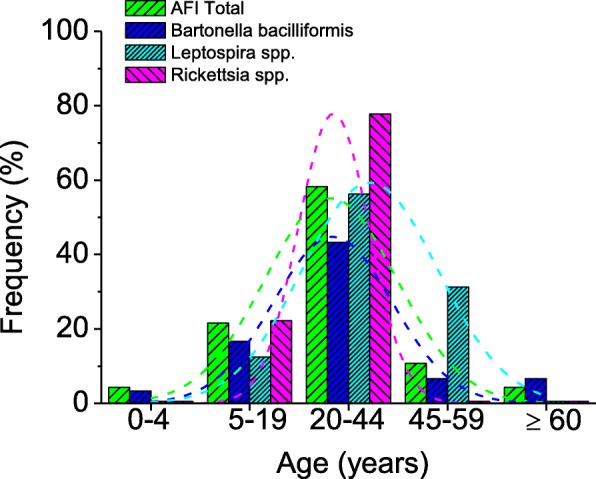
Distribution of AFI frequency with and without diagnosis of the etiological agent in the population of Puerto Maldonado-Madre de Dios, Peru. Ordered by age ranges, the curves are the Gauss models fitted

Table 1 shows AFI frequencies distributed by ages, with most patients positive to at least one of the etiologic agents being between 20 to 44 years old. However, the exhaustive analysis of frequency distributions by age and etiological agents (Fig. 2) shows that all distributions by ages correspond to Gaussian models. Patients infected with *Bartonella bacilliformis* showed a wide symmetric distribution centered in the range of 20-44 years, similar to the total population with AFI. However, the patients infected with *Leptospira* spp*.* and *Rickettsia* spp*.* showed significantly different characteristics in their distributions (|^2^, *p*<0.05) in comparison to the total population with AFI. The distribution was wide and centered between the ranges of 20-44 and 45-59 years old, indicating that adults were the most affected when infected with *Leptospira* spp. Meanwhile, when *Rickettsia* spp. was responsible for AFI, the distribution was narrower and centered in the group of young people, between 5-19 and 20-44 years old (Fig. 2). Finally, the distribution of AFI bacterial etiology by sex did not show any significant differences. Only a slight tendency of a greater distribution of *Leptospira* in males can be observed with a frequency of 62.5% (CI95%: 38.6-81.5) (Table 1).

**Table 1:** Demographics in patients with *Bartonella bacilliformis, Leptospira* spp. and *Rickettsia* spp. from Puerto Maldonado- Madre de Dios, Peru

	AFI Total		*Bartonella bacilliformis*				*Leptospira* spp*.*				*Rickettsia* spp*.*			
	*N* = 139	(%)	*N* = 30	(%)	CI 95% (%)	OR	*N* = 16	(%)	CI 95% (%)	OR	*N* = 9	(%)	CI 95% (%)	OR
Age (years)														
0–4	6	4.3	1	3.3	0.6–16.7	0.764	0	0	0.0–19.4	0.000	0	0	0.0–29.9	0.000
5–19	30	21.6	5	16.7	7.3–33.6	0.727	2	12.5	3.5–36.0	0.519	2	22.2	6.3–54.7	1.038
20–44	81	58.3	20	66.7	48.8–80.8	1.432	9	56.2	33.2–76.9	0.921	7	77.8	45.3–93.7	2.506
45–59	16	11.5	2	6.7	1.9–21.3	0.549	5	31.2	14.2–55.6	3.494	0	0	0.0–29.9	0.000
≥ 60	6	4.3	2	6.7	1.9–21.3	1.583	0	0	0.0–19.4	0.000	0	0	0.0–29.9	0.000
Gender														
Female	63	45.3	14	46.7	30.2–63.9	1.056	6	37.5	18.5–61.4	0.724	5	55.6	26.7–81.1	1.508
Male	76	54.7	16	53.3	36.1–69.8	0.947	10	62.5	38.6–81.5	2.011	4	44.4	18.9–73.3	0.663

### Clinical presentation

In this study, the most frequent symptom among all positive patients was a headache in 79.9% of all cases, followed by myalgias and arthralgias with 69% and 64%, respectively. The clinical presentation in patients positive for *Bartonella bacilliformis*, *Leptospira* spp. and *Rickettsia* spp*.*, was similar. In patients infected with *B. bacilliformis*, headaches were present in 73.3%, followed by myalgias and arthralgias in 60% of samples. Similarly, patients positive for *Leptospira* spp, had headaches (87.5%), myalgias (68.8%) and arthralgias (62.5%) as their most common symptoms (Table 2).

**Table 2:** Clinical symptoms in patients with positive diagnostic for *Bartonella bacilliformis, Leptospira* spp*.* and *Rickettsia* spp. from Puerto Maldonado-Madre de Dios, Peru

Signs and symptoms	AFI Total		*Bartonella bacilliformis*				*Leptospira* spp*.*				*Rickettsia* spp*.*			
	*N* = 139	%	*N* = 30	%	CI 95% (%)	OR	*N* = 16	%	CI 95% (%)	OR	*N* = 9	%	CI 95% (%)	OR
Headaches	111	79.9	22	73.3	55.6–85.8	0.694	14	87.5	64.0–96.5	1.766	3	33.3	12.1–64.6	0.126
Myalgia	96	69.1	18	60.0	42.3–75.4	0.672	11	68.8	44.4–85.8	0.985	2	22.2	6.3–54.7	0.128
Arthralgia	89	64.0	18	60.0	42.3–75.4	0.843	10	62.5	38.6–81.5	0.936	1	11.1	2.0–43.5	0.070
Loss of appetite	48	34.5	7	23.3	11.8–40.9	0.577	5	31.2	14.2–55.6	0.862	0	0	0.0–29.9	0.000
Retroocular pain	48	34.5	9	30.0	16.7–47.9	0.812	6	37.5	18.5–61.4	1.138	0	0.0	0.0–29.9	0.000
Nausea	40	28.8	10	33.3	19.2–51.2	1.238	4	25.0	10.2–49.5	0.825	0	0.0	0.0–29.9	0.000
Chills	22	15.8	7	23.3	11.8–40.9	1.619	2	12.5	3.5–36.0	0.760	0	0.0	0.0–29.9	0.000
Vomits	11	7.9	2	6.7	1.9–21.3	0.831	2	12.5	3.5–36.0	1.662	0	0.0	0.0–29.9	0.000
Dizziness	10	7.2	2	6.7	1.9–21.3	0.921	1	6.2	1.1–28.3	0.860	0	0.0	0.0–29.9	0.000
Rash	10	7.2	2	6.7	1.9–21.3	0.921	1	6.2	1.1–28.3	0.860	0	0.0	0.0–29.9	0.000
Sorethroat	9	6.5	3	10.0	3.5–25.6	1.605	2	12.5	3.5–36.0	2.064	0	0.0	0.0–29.9	0.000
Photophobia	9	6.5	3	10.0	3.5–25.6	1.605	1	6.2	1.1–28.3	0.963	0	0.0	0.0–29.9	0.000
Abdominal pain	8	5.8	1	3.3	0.6–16.7	0.565	2	12.5	3.5–36.0	2.340	1	11.1	2.0–43.5	2.047
Cough	6	4.3	2	6.7	1.9–21.3	1.583	1	6.2	1.1–28.3	1.478	0	0.0	0.0–29.9	0.000
Pallor	5	3.6	2	6.7	1.9–21.3	1.914	0	0.0	0.0–19.4	0.000	0	0.0	0.0–29.9	0.000
Diarrhea	5	3.6	2	6.7	1.9–21.3	1.914	1	6.2	1.1–28.3	1.787	0	0.0	0.0–29.9	0.000
Conjunctival injection	5	3.6	2	6.7	1.9–21.3	1.914	1	6.2	1.1–28.3	1.787	0	0.0	0.0–29.9	0.000
Rhinorrhea	4	2.9	1	3.3	0.6–16.7	1.164	0	0.0	0.0–19.4	0.000	0	0.0	0.0–29.9	0.000
Shortness of breath	3	2.2	0	0.0	0.0–11.4	0.000	0	0.0	0.0–19.4	0.000	0	0.0	0.0–29.9	0.000
Dysuria	2	1.4	1	3.3	0.6–16.7	2.362	0	0.0	0.0–19.4	0.000	0	0.0	0.0–29.9	0.000
Fatigue	2	1.4	0	0.0	0.0–11.4	0.000	0	0.0	0.0–19.4	0.000	0	0.0	0.0–29.9	0.000
Jaundice	2	1.4	1	3.3	0.6–16.7	2.362	0	0.0	0.0–19.4	0.000	0	0.0	0.0–29.9	0.000
Seizures	1	0.7	1	3.3	0.6–16.7	4.759	0	0.0	0.0–19.4	0.000	0	0.0	0.0–29.9	0.000

## Discussion

Acute febrile illness (AFI) is a common infectious syndrome in the Peruvian Amazon basin, caused by various bacterial and viral pathogens. In recent years, cyclic weather phenomena such as El Niño-Southern Oscillation have been implicated in the increasing incidence and resurgence of some neglected infections responsible for AFI in Peru [6–8, 19, 26]. However, due to the lack of sensitive and specific on-site diagnostic methods, most of these pathogens remain poorly characterized during outbreaks, leading to an underestimation of the real disease burden [27].

**Fig. 3:**
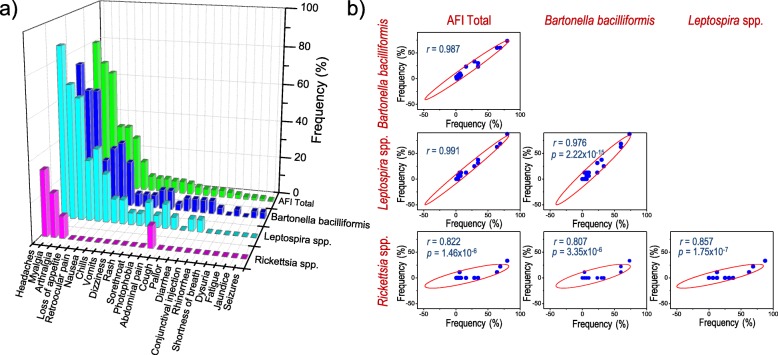
Analysis of signs and symptoms for the AFI diagnosed with an etiological agent. **a** Frequencies distribution of the signs and symptoms. **b** Scatter matrices for the correlation of signs and symptoms. Pearson coefficient (*r*) and associated probability (*p*) to the correlation

During this study, we were able to collect and analyze the clinical presentation of patients with AFI caused by three different bacterial etiologies. As shown in Fig. 3a, the frequency of symptoms of patients infected with *Bartonella bacilliformis*, *Leptospira* spp. and *Rickettsia* spp., were ordered in a descending frequency and compared with the signs and symptoms of all patients with AFI that includes those positive to arboviruses from our previous study. The analysis of the matrices shows that the frequency distributions were statistically different (χ^2^, *p*<0.05). In the case of patients with AFI caused by *Bartonella bacilliformis* and *Leptospira* spp, a bimodal distribution of symptoms was observed in comparison to the unimodal matrix of total number patients with AFI; whereas the matrix of the AFI caused by *Rickettsia* spp. shows a unimodal array characterized by very low frequencies (Fig. 3a).

However, the correlation observed between the frequencies of the signs and symptoms for the total number of patients with AFI and those infected with *Bartonella bacilliformis* and *Leptospira* spp. were very positive. Their association was 98.7% and 99.1% respectively; and in the case of AFI caused by *Rickettsia* spp. *e*, it was positive but with a low correlation of 82.2% (Fig. 3b). These results highlight the great challenge clinicians face when making an etiological diagnosis without specific diagnostic tools among patients living in regions endemic to both bacterial and viral etiologies of AFI.

Madre de Dios is endemic to dengue virus, oropouche virus, malaria and leptospirosis [6, 7, 10–13, 17]. Other bacterial pathogens such as *Rickettsia* spp. and *B. bacilliformis* have been implicated as etiological causes of AFI; however, few reports describe the burden these entities account for in this region [10, 19, 20]. Recently a meta-analysis [29] describes in Colombia the presence of Leptospira and Rickettsia, with frequencies in the ranges of 14-27% and 2-6% respectively. In our previous study, we identified the presence of arboviruses including dengue, chikungunya, Zika, and oropouche, in 139 patients with AFI from Puerto Maldonado in Madre de Dios [25, 29]. We were able to identify at least one of these viruses in 29.5% (41/139) of cases. In the present study, we have analyzed the same samples to identify *Bartonella bacilliformis*, *Leptospira* spp. and *Rickettsia* spp molecularly. Interestingly, we were not able to detect co-infections between the identified viruses from our previous study and the bacterial pathogens identified in this study. Furthermore, we were able to identify *B. bacilliformis* in 21.6% (30/139) of samples, *Leptospira* spp*.* in 11.5% (16/139) and *Rickettsia* spp. in 6.5% (9/139) cases, without any co-infections between these bacteria.

*Bartonella bacilliformis* is endemic to many regions in Peru and its neighboring countries [30]. The on-site diagnosis of this infection is mainly based on the clinical suspicion and extensive use of peripheral blood smears given the intraerythrocytic nature of the bacteria. However, the sensitivity of this method has been reported to be low, between 24%-36% [31, 32], in comparison to molecular methods with reported sensitivity and specificity of 100% [33]. In contrast to the national reports that found 2 positive cases in 2016 in Madre de Dios [34], we molecular identified *Bartonella bacillliformis* in 30 patients (21.6%) with acute febrile illness during our study 3-month study period. This contrasting frequency may be due to the molecular method employed and highlights the importance of implementing these tools to enhance national surveillance programs.

Leptospirosis is a widespread, underreported and, prevalent zoonotic disease with no reliable global incidence data. A model made by the World Health Organization’s (WHO) about the burden of the disease estimated that there were 873 000 cases worldwide annually with 48 600 deaths [35]. In Peru, the incidence of leptospirosis is approximately 2 000 cases annually. In 2016, 2 063 cases of leptospirosis were reported, and nearly 50% (*n*=1002) corresponded to patients from Madre de Dios [36].

In the present study, we were able to identify 16 (11,5%) cases of *Leptospira* spp. infection from 139 samples of patients with AFI. The clinical course of leptospirosis is variable and most cases are self-limited or subclinical, while less frequent cases are severe and potentially fatal [37]. The most common symptoms found in this group were headaches (87,5%), myalgias (68,8%), and arthralgias (62,5%) that corresponds the clinical presentation of mild leptospirosis. This presentation may be to the fact that all the studied samples came from outpatient health care centers. Furthermore, a case-control study found that risk factors for the development of severe leptospirosis included a delay of more than 2 days following the start of symptoms in the initiation of antibiotic therapy [38], highlighting the importance of accurate and timely diagnosis of this emergent bacterial disease.

In our study, we were able to detect 9 cases (6.5%) of *Rickettsia* spp. via real-time PCR. This is a fairly high frequency considering that *Rickettsia* spp. is an intracellular organism and some studies report a low sensitivity using molecular methods [39]. It is important to consider the results of some serological studies that suggest a high level of transmission of spotted fever group rickettsiae (SFGR) and of the Tifus group rickettsiae (TGR) in the Peruvian Amazon, 46.3% and 10.3% respectively. More studies identifying Rickettsia species are needed to assess their role in the AFI and quantify the impact of the associated disease burden [40, 41]. Our findings also show that the clinical presentation of this infection is non-specific when compared to patients with AFI, following a pattern reported in previous studies [42, 43]. Surprisingly, no co-infections were identified in patients infected with *Rickettsia* spp.

The main limitation of this study is our inability establish causality between the identified bacteria and the clinical presentation. However, due to the similar symptoms registered across all groups, we can still conclude that molecular diagnostic tests should be mandatory for the etiological diagnosis of AFI. Finally, as we only studied cases in the outpatient setting, more severe cases that required hospitalization might not have been included in our analysis.

## Conclusion

Our study series have shown that arboviruses (29.5%), *B. bacilliformis* (21.6%), *Leptospira* spp. (11.5%) and *Rickettsia* spp. (6.5%) accounted for the main etiologies of AFI. Our analysis of their clinical presentation, further shows the importance of implementing more sensitive and specific on-site diagnostic tools in the national surveillance programs, as this study confirms that the un-specificity of signs symptoms is not only associated with arboviral infections [25], but also with the clinical presentation of endemic bacterial infections.

## Abbreviations

AFI: Acute febrile illnesses
bp: Base pairs
DNA: Deoxyribonucleic acid
PCR: *Polymerase chain reaction*
